# OVX033, a nucleocapsid-based vaccine candidate, provides broad-spectrum protection against SARS-CoV-2 variants in a hamster challenge model

**DOI:** 10.3389/fimmu.2023.1188605

**Published:** 2023-06-19

**Authors:** Charlotte Primard, Elodie Monchâtre-Leroy, Judith Del Campo, Séverine Valsesia, Elsa Nikly, Marion Chevandier, Franck Boué, Alexandre Servat, Marine Wasniewski, Evelyne Picard-Meyer, Thomas Courant, Nicolas Collin, Francisco J. Salguero, Alexandre Le Vert, Delphine Guyon-Gellin, Florence Nicolas

**Affiliations:** ^1^ Osivax, Lyon, France; ^2^ ANSES, Laboratory for Rabies and Wildlife, Malzéville, France; ^3^ VFI, Plan-Les-Ouates, Switzerland; ^4^ UKHSA, Salisbury, United Kingdom

**Keywords:** recombinant nucleocapsid, universal vaccine, broad-spectrum protection, cross-reactive immune responses, lung histopathology, lung viral load, T cell immunity, sarbecovirus vaccine

## Abstract

Spike-based COVID-19 vaccines induce potent neutralizing antibodies but their efficacy against SARS-CoV-2 variants decreases. OVX033 is a recombinant protein composed of the full-length nucleocapsid (N) protein of SARS-CoV-2 genetically fused to oligoDOM^®^, a self-assembling domain which improves antigen immunogenicity. OVX033 including N as an antigenic target is proposed as new vaccine candidate providing broad-spectrum protection against sarbecoviruses. OVX033 demonstrated its ability to trigger cross-reactive T cell responses and cross-protection against three variants of SARS-CoV-2 (B.1 Europe, Delta B.1.617.2, and Omicron B.1.1.529) in a hamster challenge model, as evidenced by lower weight loss, lower lung viral loads, and reduced lung histopathological lesions.

## 1 Introduction

The severe acute respiratory syndrome coronavirus 2 (SARS-CoV-2) was responsible for coronavirus disease 2019 (COVID-19) pandemic. The disease already caused the death of several millions of people and posed major economic challenges as well as public health crisis globally (1). The burden of SARS-CoV-2 has been reduced by spike-based vaccines, which trigger humoral and cellular responses against the surface protein. Specifically, anti–SARS-CoV-2 neutralizing antibodies are considered as a correlate of protection (2). However, this protection is known to decrease (3) with the emergence of variants of concern (VoCs) with multiple mutations in key neutralizing epitopes that evade the host’s adaptive responses. There is also a significant waning in the humoral response over time, particularly among immunocompromised people or individuals over 65 years old. This highlights the need for longer lasting and more broadly protective vaccines. Among the coronavirus proteins capable of eliciting cross-reactive responses, the structural nucleocapsid (N) protein is of major interest, as one of the most abundant proteins produced during viral replication and considering its high degree of homology across sarbecoviruses (4–6). The N protein is a prominent target of SARS-CoV-2 specific T-cell responses during COVID-19, and the role of T-cell immunity in controlling SARS-CoV-2 infection is now widely recognized (7). SARS-CoV-1 N-specific memory T cells of people infected during the 2002–2003 SARS outbreak were shown to cross-react with the N protein of SARS-CoV-2 (8), as the two N proteins share 90% homology (4). SARS-CoV-2 N-specific CD8^+^ T cells have been linked to protection against severe disease, control of viral replication, and maintained antiviral efficacy against multiple variants (Alpha, Beta, Gamma, and Delta) at least 6 months after infection (9). N-specific antibody responses also correlated with viral clearance in the lungs, by eliciting NK-mediated and antibody-dependent cellular cytotoxicity (ADCC) against infected cells (10). Hence, generating immune responses against N is of critical importance for developing broad-spectrum vaccines.

OVX033 is a recombinant vaccine candidate including the full-length nucleocapsid antigen of SARS-CoV-2 virus (Wuhan original strain). N antigen was genetically fused to OVX313 sequence (oligoDOM^®^), OSIVAX’s self-assembling domain which improves antigen immunogenicity (11). Contrarily to spike-based vaccines that aim to generate antibody responses neutralizing the circulating SARS-CoV-2 viruses, OVX033 N-based vaccine aims to kill the infected cells, thus limiting the infection and the disease symptoms. As N is well conserved across sarbecoviruses, the OVX033 vaccine is assumed to protect similarly against various sarbecovirus strains. In this paper, we present the first results of cross-protection provided by OVX033 (alone, or adjuvanted by a squalene-in-water emulsion containing cholesterol and QS21 saponin, SQ adjuvant) against various SARS-CoV-2 VoCs in a hamster challenge model, as well as cross-reactive immunogenicity data against these variants.

## 2 Methods

### 2.1 OVX033 vaccine

**Figure 1:**
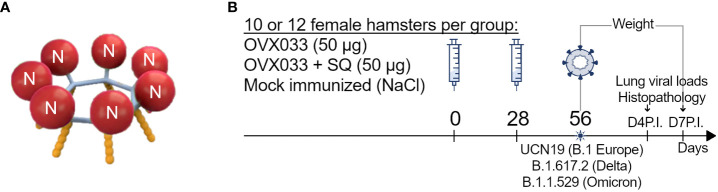
**(A)** Schematic representation of OVX033 vaccine candidate. OVX033 is a recombinant multimeric protein composed by the full-length Nucleocapsid (N) of the SARS-CoV-2 virus (Wuhan original strain), genetically fused to the self-assembling sequence OVX313 (oligoDOM^®^). **(B)** Study design: Hamsters were immunized twice 28 days apart with OVX033, OVX033 adjuvanted with SQ (OVX033+SQ), or saline. The animals were challenged by intranasal instillation of 10^4^ TCID_50_ of one of the three SARS-CoV-2 strains, B.1 Europe (UCN19), Delta (B.1.617.2), or Omicron (B.1.1.529). After challenge, the animals were monitored daily for body weight up to 7 days and euthanized at either 4 or 7 days post-inoculation to measure viral loads in lungs and for lung histopathological analyses.

OVX033 is a recombinant multimeric protein, composed of the full-length nucleocapsid of the SARS-CoV-2 virus (Wuhan original strain), genetically fused to the self-assembling sequence OVX313 [oligoDOM^®^ (11)], as illustrated in **Figure 1A**. OVX313, whose sequence is detailed in Del Campo et al. (12), is a 55-amino acids sequence, hybrid of the C-terminal fragments of two avian C4bp α chain sequences, that naturally oligomerizes into heptamers. OVX033 vaccine was produced in *Escherichia coli* and highly purified (Osivax, France). SDS-PAGE purity of the protein was > 90%, and the residual host endotoxin (≤ 9 EU/mg) and DNA (≤ 29 ng/mg) levels were very low. OVX033 was tested unadjuvanted or formulated with SQ adjuvant, a squalene-in-water emulsion containing cholesterol and QS21 saponin from *Quillaja saponaria*, developed by the Vaccine Formulation Institute (VFI, Switzerland) (13).

### 2.2 SARS-CoV-2 viruses

SARS-CoV-2 strain UCN19 (B.1 Europe) was isolated in March 2020 during the active epidemic, from naso-pharyngeal flocked swabs obtained from a patient at the University Hospital of Caen, Normandy, France (GISAID reference EPI_ISL_666870) (14). SARS-CoV-2 Lineage B.1.617.2, Delta was provided by EVA-GLOBAL PARTNER (clade 21A, strain SARS-CoV-2/2021/FR/0610 GISAID reference EPI_ISL_2838050). SARS-CoV-2 Lineage B.1.1.529, Omicron from clade 21K, or BA.1, was provided by VirNext, France (GISAID reference EPI_ISL_7360393). All viral strains were amplified on African green monkey kidney cells (VERO E6).

### 2.3 Ethics statement

The experimental protocols comply with the directive 2010/63/CE of the European Parliament and of the council of 22 September 2010 (15). The protocols of challenges and immunogenicity conducted in hamsters were approved respectively by the Anses/ENVA/UPEC and the VetAgro Sup ethic committees and authorized by the French Ministry of Research (APAFIS#33930-2021111716444848 v2 and APAFIS #16460-2018080819285585).

### 2.4 Animal experimental designs

Eight-week-old female Syrian Golden hamsters (*Mesocricetus auratus*, strain RjHan : AURA, Janvier Labs, France) were housed in cages of two individuals with environmental enrichment, food, and water provided ad libitum. They were randomly assigned in the experimental groups and subgroups at the start of the study to roughly obtain equivalent weight. Hamsters were immunized twice 28 days apart, by intramuscular (IM) injections with 50 µg of OVX033, OVX033 adjuvanted with SQ, or NaCl 0.9% as negative control. Twenty-eight days after the last immunization, hamsters were infected by intranasal (IN) instillation of 40 µl containing 10^4^ median tissue culture infectious dose (TCID_50_) of one of the 3 SARS-CoV-2 strains, B.1 Europe (UCN19), Delta (B.1.617.2), or Omicron (B.1.1.529) as described in Bessière et al. (16) and illustrated in **Figure 1B**. Animals were monitored daily for up to 7 days for body weight. Lungs were collected on days 4 and 7 post-inoculation (P.I.), and either stored frozen as previously described (14) for viral load titration and for TaqMan SARS-CoV-2 RT-qPCR assays, or stored in 10% neutral formalin for histological analyses.

For immunogenicity study, hamsters were immunized twice as described above. Serum was collected at D0 and D42, final time point, for evaluation of anti-N IgG titers by enzyme-linked immunosorbent assay (ELISA). A vascular flush was done under anaesthesia followed by spleen and lung sampling for T cell responses evaluation by IFNγ enzyme-linked immunospot (ELIspot) assay.

### 2.5 SARS-CoV-2 virus titration

SARS-CoV-2 titrations from viral inoculum before and after infection and from lung samples were performed by plaque assay on VERO E6 in cell culture medium (DMEM) supplemented with 10% FCS and 1% penicillin/streptomycin. Disposable 96-well microplates were seeded with 20,000 cells per 200 µl per well for 24h at 37°C in a humid chamber containing 5% CO_2_, before virus inoculation. The supernatant fluid was removed from each well. For the viral reference strain, a 10-fold serial dilution from 10^-2^ to 10^-11^ was performed, and for the tested samples, a fourfold serial dilution from 10^-1^ to 10^-7.6^ was performed, in cell complete culture medium (DMEM). Six uninfected wells were used as independent negative controls. Fifty microliters of each dilution were added in six consecutive wells. After adsorption of the virus for 60 min at 37°C in a humid chamber containing 5% CO2, 200 µl of fresh growth medium were added to each well. The plates were incubated under the same conditions for 72h. Virus titers were determined by using the Spearman-Kärber formula and the titers were expressed in TCID_50_/ml.

### 2.6 RNA extraction and TaqMan RT-qPCR

RNA extraction from tissue homogenates was performed as described in (14) on AVL Lysis buffer (Qiagen, Courtaboeuf, France) with 2.7% Triton X-100 (MP Biomedicals, Illkirch, France) to inactivate potential infectious status of samples by SARS-CoV-2. A negative control RNA extraction was performed for each set of 12 samples tested. TaqMan RT-qPCR was performed with Quantitect Probe RT-PCR kit (Qiagen, Courtaboeuf, France) with E-Sarbeco primer-probes targeting the envelope protein gene (E gene), on the thermocycler Rotor Gene Q MDx (Qiagen, Courtaboeuf, France). Absolute quantification (expressed in number of copies/µl) was undertaken using a standard curve based on six 10-fold dilutions of a SARS-CoV-2 RNA titrating 3.10^6^ E gene copies/µl of RNA. A threshold setting (Ct) of 0.05 was used as the reference for each RT-qPCR assay.

### 2.7 Lung histological analyses and *in-situ* hybridization

Lung lobes fixed in 10% neutral-buffered formalin were processed into paraffin wax, cut in 4 µm sections and stained with hematoxylin and eosin (H&E) before examination by light microscopy (Hamamatsu NanoZoomer S360) and a subjective evaluation by a qualified pathologist (UKHSA, Salisbury, United Kingdom). The pathologist was blinded to treatment and group details and the slides were randomized prior to examination in order to prevent bias (blind evaluation). Tissue sections were scanned by a Hamamatsu NanoZoomer S360 and viewed with NDP.view2 software. A semi-quantitative, subjective scoring system previously described [(17) and **Supplementary Table S1**] was used to evaluate the severity of lesions observed in the lung. In addition, the percentage of area with pneumonia was calculated using digital image analysis (Nikon-NIS-Ar).

Lung samples were also stained using the RNAscope *in-situ* hybridization (ISH) technique to identify the SARS-CoV-2 virus RNA as previously described (18). Digital image analysis was carried out to calculate the total area of the lung section positive for viral RNA.

### 2.8 Enzyme linked immunosorbent assay

ELISA plates (Nunc MaxiSorp^®^) were coated overnight with 1.5 µg/ml of recombinant N protein of SARS-CoV-2 (Wuhan original strain as in OVX033) without oligoDOM® domain, produced in *Escherichia coli* and purified by chromatography by Osivax. After 1h saturation with 3% Bovine Serum Albumin (Merck Life Science, France) in PBS at 25°C, serial twofold dilutions of serum (starting dilution 1/200) were incubated for 2h. Goat anti-hamster IgG-HRP (Thermo Fisher Scientific, France) was added for 1h at 25°C, followed by tetramethylbenzidine (TMB) substrate (Interchim, France) and then stop reagent (Merck Life Science, France). N-specific antibody levels were expressed as endpoint dilution titer, defined as the reciprocal of the highest analytic dilution that gives a reading threefold over the mean O.D. 450 value of pre-immune serum at the 1/100 dilution. Before statistical analysis, a log transformation was performed on values.

### 2.9 IFNγ enzyme-linked immunospot assay

Using Hamster IFNγ Precoated ELISPOT kits (Mabtech, Sweden), 2.5 × 10^5^ splenocytes and lung cells were stimulated with Wuhan, Delta (B.1.617.2) and Omicron (B.1.1.529) peptide pools at 2 μg/ml (15-mers overlapping by 11 amino acids, JPT, Berlin, Germany) for 20h at 37°C, 5% CO_2_. Concavalin A (Merck Life Science, France) was used as a positive control and unstimulated cells as negative control. Spots were detected according to the manufacturer’s instructions and air-dried plates were read using an automated ELISPOT reader (Mabtech ASTOR ELISpot reader). The number of peptide-reactive cells was represented as spot-forming units (SFUs) per 1 × 10^6^ cells (background subtracted).

### 2.10 Statistical analyses

Graphic representations and statistical analyses were performed with Prism 9 (GraphPad Software Inc., MA, USA), using non-parametric Kruskal–Wallis test followed by Mann & Whitney tests between treated and mock-immunized groups. Differences were considered significant if the p value was < 0.05:*, < 0.01:**.

## 3 Results

**Figure 2:**
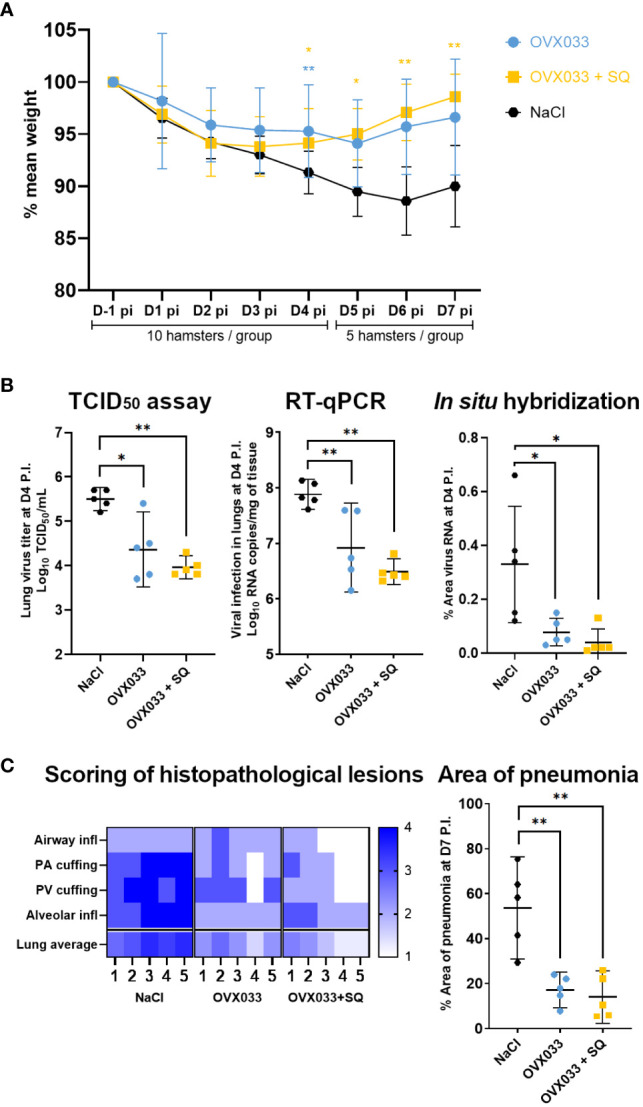
Immunization with OVX033 alone or adjuvanted with SQ protected against severe disease after challenge with SARS-CoV-2 B.1 Europe in a hamster model. Syrian Hamsters were immunized twice with OVX033, OVX033+SQ or saline, then challenged intranasally with 10^4^ TCID_50_ of SARS-CoV-2 B.1 Europe (UCN19). **(A)** Percent weight change (mean ± SD), significance was measured at each time point. **(B)** Viral infection in lungs 4 days P.I., evaluated by TCID_50_ assay (left panel), RT-qPCR (middle panel) and *in-situ* hybridization (right panel) characterized by the percentage of area positively stained against viral RNA in lung sections. Individual values and mean ± 95% CI. **(C)** Lung histopathological analysis 7 days P.I. Left panel: heatmap showing severity for each lung histopathology parameter and animal (from 1 to 5). Airway inflammation/necrosis, peri-airway cuffing, peri-vascular cuffing, alveolar inflammation/necrosis. Scoring criteria are detailed in **Supplementary Table S1**. Right panel: percentage of area with pneumonia as determined by image analysis of lung sections treated with hematoxylin and eosin (individual values and mean ± 95% CI). Significance was measured by Kruskal Wallis test, followed by Mann & Whitney tests between treated groups and mock-immunized group. *p < 0.05 and **p < 0.01.

The protective efficacy of OVX033 vaccine was evaluated in a hamster model of SARS-CoV-2 infection. Golden Syrian hamsters are highly susceptible and responsive to the SARS-CoV-2 virus and develop disease symptoms that closely resemble the COVID-19 pathogenesis (14, 17, 19, 20). Hamsters immunized twice with OVX033 alone; OVX033 adjuvanted with SQ or saline were intranasally challenged with SARS-CoV-2 (B.1 Europe) and monitored for up to 7 days for body weight (**Figure 1B**). Mock-immunized hamsters lost around 10% weight by day 6 P.I.. Immunized hamsters had significantly less weight loss at D4 P.I., and from 4 to 7 days P.I. for the OVX033+SQ group (**Figure 2A**). Viral titers were determined at D4 P.I. in lungs. Animals immunized with OVX033 alone or adjuvanted had significantly lower lung viral loads 4 days P.I. than controls as assessed by TCID_50_ assay, RT-qPCR, and *in-situ* hybridization on lung sections (**Figure 2B**). Animals immunized with OVX033 alone displayed some interindividual heterogeneity, while the viral loads in animals immunized with OVX033+SQ were more homogeneous within the group (**Figure 2B**). Besides, histopathology analyses showed significantly less inflammation in lungs of immunized animals vs controls (**Figure 2C** and **Supplementary Figure 1**), notably characterized by lower alveolar inflammatory cell infiltration (primarily macrophages and neutrophils, with some lymphocytes and plasma cells) and lower presence of lymphoplasmacytic perivascular infiltration (perivascular cuffing). Finally, immunized animals had significantly lower pneumonia incidence than the control group (**Figure 2C**). OVX033 alone being very efficient to protect from lung lesions, the effect of the adjuvant was marginal on these parameters. Leveraging these promising results providing a proof-of-protection of OVX033 against the SARS-CoV-2 B.1 variant, the level of protection against additional VoCs was further explored.

**Figure 3:**
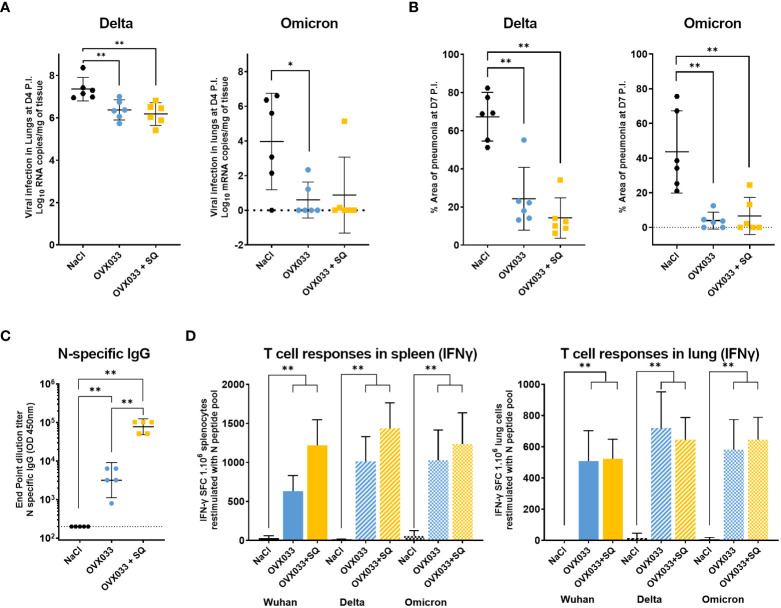
Immunization with OVX033 alone or adjuvanted with SQ protected hamsters against severe disease after challenge with SARS-CoV-2 VoC Delta (B.1.617.2) or Omicron (B.1.1.529), and induced N-specific humoral and cross-reactive cellular responses. **(A)** Lung viral load assessed by RT-qPCR 4 days post-challenge with Delta (left panel) or Omicron (right panel). **(B)** Mean percentage of area with pneumonia in the lung 7 days post-challenge with Delta (left panel) or Omicron (right panel) as determined by image analysis. Individual values and mean ± 95% CI. (**C**, **D**) Immunogenicity of OVX033 and OVX033+SQ in hamsters, 14 days after the second immunization. **(C)** N-specific seric IgG titers. Individual titers and GMT ± 95% CI. Significance was measured by Mann & Whitney tests on log-transformed data. **(D)** T-cell responses evaluated in spleen and lungs by IFNγ ELISpot. Histograms represents the mean ± SD number of IFNγ spot forming units (SFUs) among splenocytes or cells isolated from lungs, after overnight restimulation with N peptide pools from Wuhan, Delta or Omicron (15-mers overlapping by 11 amino acids). Significance was measured by Kruskal–Wallis test, followed by Mann & Whitney tests between treated groups and mock-immunized group. *p < 0.05 and **p < 0.01.

Hamsters immunized twice as previously described were challenged with SARS-CoV-2 variants B.1.617.2 (Delta) or B.1.1.529 (Omicron), (**Figure 1B**). Delta infection caused up to 6% weight loss in mock-immunized animals, while immunization reduced weight loss, with a significant effect at D6 P.I. for OVX033+SQ (data not shown). Infection with Omicron did not cause any weight loss (data not shown), as previously described (21, 22). Lung viral loads were significantly lower at D4 P.I. in immunized groups compared with negative control as assessed by RT-qPCR (**Figure 3A**) and TCID_50_ assay (heterogeneously from 1 to 2 logs, **Supplementary Figure 2A**). Finally, immunization with OVX033 alone or OVX033+SQ strongly reduced pulmonary damages following both Delta and Omicron infections, as evidenced by significant difference in the surface areas of pneumonia in lungs 7 days P.I. (**Figure 3B**), and a decrease in the ranking of multiple criteria of lung tissue damage (**Supplementary Figures 2B, C**).

OVX033 immunogenicity was finally evaluated in hamsters, 14 days after the second IM immunization with OVX033 alone or adjuvanted with SQ. All OVX033-immunized hamsters had high N-specific IgG titers as measured by ELISA (**Figure 3C**). The titers were higher by one to two log(s) when OVX033 was adjuvanted with SQ. IFNγ producing cells were quantified in spleen and lungs using ELISpot, after stimulation with peptide pools (15-mers overlapping by 11 amino acids) covering the full-length N sequences of Wuhan, Delta, or Omicron SARS-CoV-2 variants. IFNγ T-cell responses were almost undetectable in mock-immunized animals but were significantly increased in animals immunized with OVX033 or OVX033+SQ, both in spleen and lungs (**Figure 3D**). The number of N-specific SFUs measured in the spleen was slightly superior for OVX033+SQ group compared with OVX033 alone. In lungs, both groups had comparable elevation of N-specific SFUs. Finally, T cells from spleen and lungs responded equivalently to stimulation with N peptides from Wuhan, Delta and Omicron, demonstrating the cross-reactivity of the cellular immune response triggered by OVX033.

## 4 Discussion

Developing a broad-spectrum SARS-CoV-2 vaccine remains a priority due to the regular emergence of new variants that escape both convalescent and spike-based vaccine-induced immunity and also to get better prepared in case of a new Sarbecoviurs pandemic outbreak. Our solution to address this major challenge is a recombinant protein vaccine targeting the nucleocapsid of SARS-CoV-2, which is highly conserved among Sarbecoviruses. N is the most abundant viral protein, and one of the antigens most predominantly targeted by T cells during infection, especially in individuals with mild COVID-19 disease (4).

N-based subunit vaccines were previously evaluated in pre-clinical studies, alone (23), or most often in combination, especially with S-antigen. Adenovirus (24, 25), modified vaccinia Ankara vector (26), a replicating bacterium-vectored vaccine (27), a vesicular stomatitis virus–based vaccine (28), or mRNA (21) technologies were tested. In these studies, antigens combination improved the protection against VoCs compared with N or S-antigen single vaccines. The N-targeting adenovirus–based vaccine from Matchett et al. showed a strong protective effect in hamster and K18.hACE2 (K18) mice (23), protection involving T cells and other non-neutralizing effector mechanisms. Viral vectors are attractive tools for inducing T-cell immunity, as they enable intracellular expression of antigens. However, vectors neutralization by capsid-specific antibody responses can impair vaccine efficacy. Although recombinant protein-based vaccines do not have the limitation of vector neutralization, they are generally poor inducers of cellular immune responses. This may explain why, to our knowledge, no recombinant vaccine targeting the N protein has yet been shown to provide protection against heterologous variants.

The recombinant vaccine candidate OVX033, which targets the full-length SARS-CoV-2 N protein fused with the self-assembling oligoDOM^®^ sequence, protected hamsters from three different SARS-CoV-2 variants of concern (B.1 Europe, Delta, and Omicron), as demonstrated by significantly lower weight loss, lower viral loads in the lungs, and reduced lung tissue damage after challenge. A comparable level of lung viral load decrease, 4 days post-inoculation of Delta or Omicron variants, was observed in hamsters immunized with a Spike vaccine (BioNTech BNT162b2 mRNA vaccine, based on original strain), following the same immunisation/challenge schedule (29). In this study, the animals were challenged at the peak of the anti-S humoral response (1 month after the last vaccination) and thus when the protection is thought to be optimal, while waning rapidly afterwards (30, 31).

In parallel, OVX033 immunogenicity was evaluated in the same hamster animal model. T-cell responses triggered by OVX033 in spleen and lungs showed cross-reactivity against the three SARS-CoV-2 variants and are assumed to contribute to the protection. Indeed, N-specific CD8^+^ T cells were linked to potent antiviral phenotype and milder disease in SARS-CoV-2–infected human individuals (9). In K18 mice immunized with N-based adenovirus vaccine, CD8+ and CD4+ T cells depletion prior to challenge led to partial abrogation of protection, as assessed by higher lung viral load compared with mice with unchanged T cell counts (23). OVX033 also elicited strong anti-N humoral responses. No neutralizing activity is expected from N-specific IgG as N is an internal protein of the virus (10, 21), but Fc-mediated antibody-effector functions may play a role in antiviral control (32). Indeed, N was shown to be expressed on the surface of SARS-CoV-2 infected cells (24, 33) and N-specific antibodies were described as able to bind to SARS-CoV-2 infected cells *in vitro*, triggering ADCC (10, 34). In addition, mice adoptive transfer of N-specific sera or N-specific monoclonal antibody before challenge resulted in lower weight loss and lower viral loads in lung compared with mice treated with an unspecific serum, demonstrating the potential protective role of N specific humoral response *in vivo* (10). For OVX033, the mechanism of action is not yet elucidated, and the respective roles of N-specific T cells and antibodies in the protection against SARS-CoV-2 remain to be further investigated.

SQ-adjuvanted OVX033 vaccine showed improved immunogenicity over OVX033 alone, as demonstrated by significantly higher anti-N IgG titers and rise of IFNγ T cells in the spleen but not in lung. However, no significant difference in efficacy (body weight loss, viral loads, or histopathology) was found. These outcomes on OVX033 alone were considered sufficient to support further evaluation of this new vaccine without adjuvant in a first-in-human clinical trial.

Unlike animal models, humans are no longer naive to the N antigen. Indeed, a large part of the population has developed immunity to the N protein either (i) from SARS-COV-2 infection, as 765 million COVID-19 cases were confirmed worldwide as of April 2023 (1), (ii) from previous Betacoronavirus infection, as pre-existing T cells to SARS-CoV-2 were identified in 40 to 60% of individuals prior to SARS-COV-2 outbreak in 2020 (4, 5); and potentially (iii) from vaccination with an inactivated whole SARS-CoV-2 virus, as Sinopharm/BBIBP-CorV (35) and Sinovac/CoronaVac were largely distributed in Asia and South America, while Valneva/VLA2001 was distributed in some parts of Europe (36). In such an epidemiological context, OVX033 alone may boost pre-existing N-specific immune responses. Indeed, OVX836, Osivax’s broad-spectrum flu vaccine targeting the influenza virus nucleoprotein (NP) acts as a booster for NP-specific B- and T-cell immunity acquired through previous flu infections (37, 38). In Phase 2 clinical trial, it has been shown to reduce influenza-like illness episodes (37), demonstrating that the nucleocapsid-based vaccine OVX033, which also uses the oligoDOM^®^ technology, may provide protection in humans.

These outcomes encourage further development of the OVX033 vaccine candidate targeting the highly conserved nucleocapsid antigen, which aims to offer broad protection against current and future Sarbecovirus diseases. Indeed, in case of newly emerging sarbecovirus pandemic strain, the Spike-based vaccines would need to be updated to match with its specific Spike surface antigen. Thanks to the nucleocapsid high level of homology among sarbecoviruses, an N-based vaccine such as OVX033 may overcome these limitations and could provide an immediately available vaccine to protect against any new emerging sarbecovirus strain, while Spike-specific vaccines are being updated, produced and supply globally. Adding an adjuvant such as SQ may boost immunity and expand protection against other Betacoronavirus like MERS.

## Data availability statement

The original contributions presented in the study are included in the article/**Supplementary Material**. Further inquiries can be directed to the corresponding author.

## Ethics statement

The animal studies were reviewed by the Anses/ENVA/UPEC and the VetAgro Sup ethic committees and approved by the French Ministry of Research.

## Author contributions

Conceptualization: DG-G, FN, JC, EM-L; Methodology, investigation and data curation: CP, JC, SV, EN, MC, AS, MW, EP-M, FB, TC, AV, FN, NC, FS, EM-L, and DG-G; Supervision: EM-L, FN and DG-G. Manuscript preparation: CP, JC, FN wrote the manuscript with review and editing from all authors. All authors contributed to the article and approved the submitted version.
