# Supplementary material for: OVX033, a nucleocapsid-based vaccine candidate, provides broad-spectrum protection against SARS-CoV-2 variants in a hamster challenge model
Source: Front Immunol. 2023 Jun 19;14:1188605. doi: 10.3389/fimmu.2023.1188605 (PMC10319154; doi:10.3389/fimmu.2023.1188605)
Supplement: Supplementary file 1 [file DataSheet_1.docx]

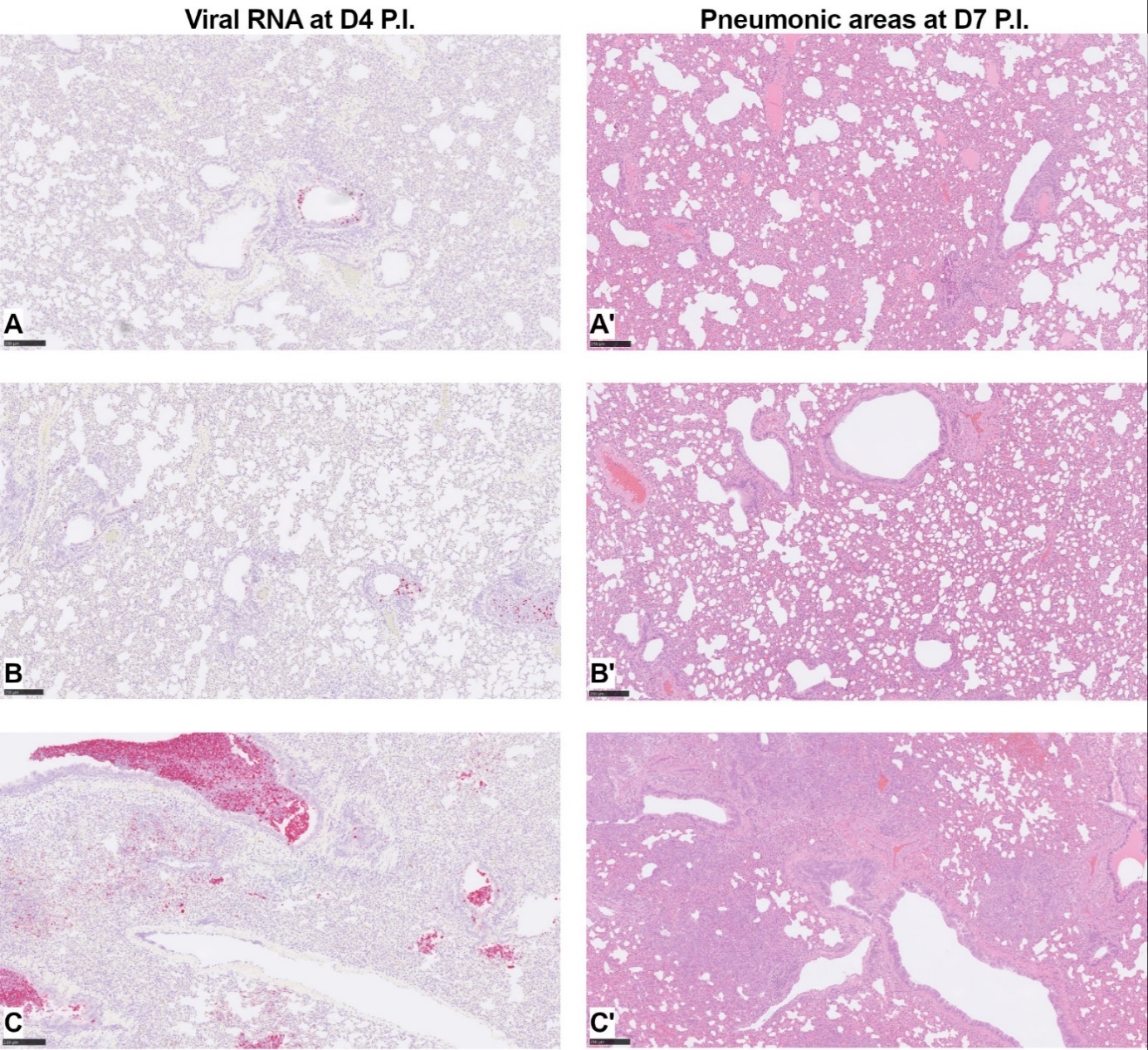


**Supplementary Figure 1.** Representative images of lung sections collected 4 or 7-days after challenge with SARS-CoV-2 (B.1 Europe). Representative images of 4-µm thick lung sections of animals challenged with B.1 Europe SARS-CoV-2 after two immunizations with OVX033 (**A, A’**), OVX033 + SQ (**B, B’**) or saline (**C, C’**). Sections of lungs collected 4 days after challenge were treated by RNAscope (an in-situ hybridisation method) to detect WT SARS-CoV-2 virus RNA (in red, **A-C**). Sections of lungs harvested 7 days after challenge were treated with hematoxylin and eosin (HE) (**A’-C’**). Scale bar 250 nm.


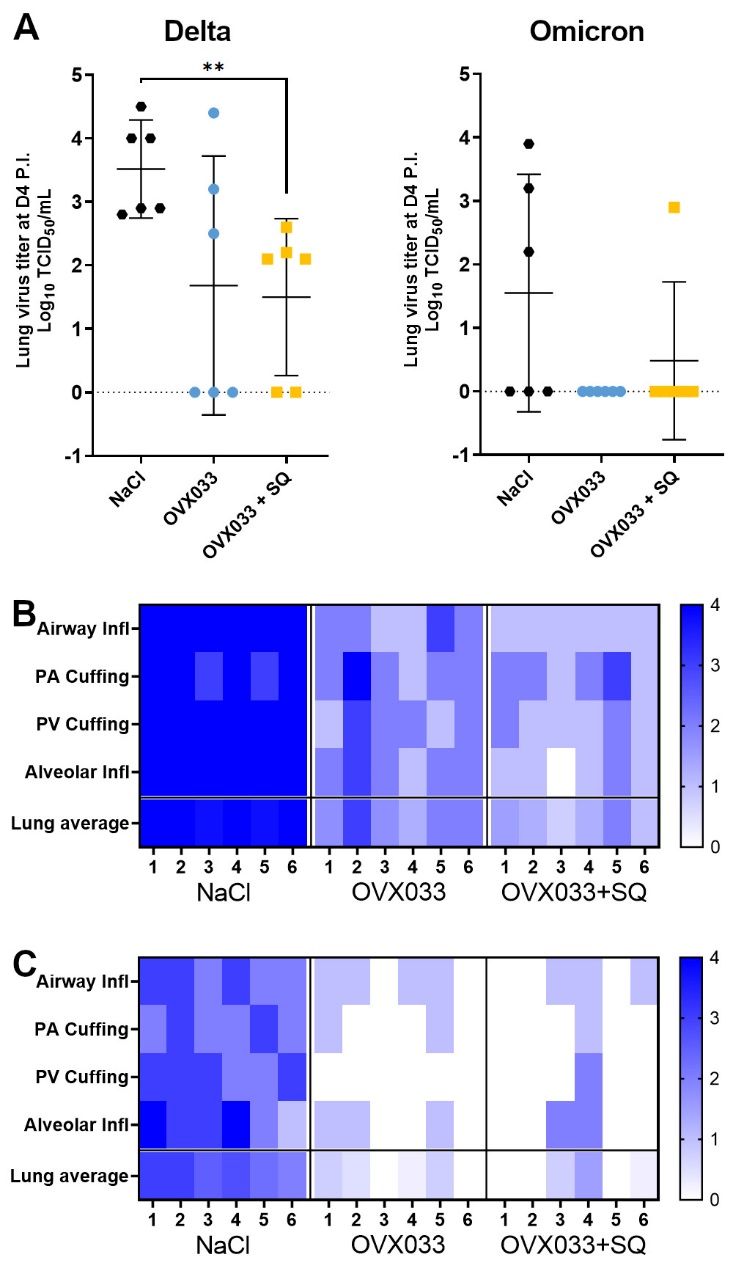


**Supplementary Figure 2.** Immunization with OVX033 or OVX033+SQ protected against severe disease after challenge with SARS-CoV-2 Delta or Omicron in hamster challenge model. Naïve hamsters were vaccinated twice 28 days apart with OVX033, OVX033 + SQ or saline. Animals were challenged by intranasal instillation of either Delta (B.1.617.2) or Omicron (B.1.1.529) SARS-CoV-2 strains, and then euthanized at either 4 or 7 days post-inoculation, to measure viral load in the lungs and for histopathological analyses. **A** Viral titers in lungs (TCID_50_/mL), 4 days after challenge with Delta or Omicron. Dot plots represent individual values and mean ± 95% CI. Significance was measured by Kruskal Wallis test, followed by Mann & Whitney tests between treated groups and mock-immunized group. **p<0.01. **B**, **C** Heatmaps showing severity for each lung histopathology parameter and animal (from 1 to 6) after challenge with **B** Delta or **C** Omicron. Airway inflammation / necrosis, peri-airway cuffing, peri-vascular cuffing, alveolar inflammation / necrosis. Scoring criteria are detailed in Supplementary Table 1.

**Supplementary Table 1.** Scoring criteria for the subjective assessment of microscopic changes in lung of hamsters infected with SARS-CoV-2

| **Lesion** | **Score 0**  **(normal)** | **Score 1**  **(minimal)** | **Score 2**  **(mild)** | **Score 3**  **(moderate)** | **Score 4**  **(marked)** |
| --- | --- | --- | --- | --- | --- |
| Airway epithelial  degeneration/necrosis and/or inflammatory cell infiltration with or  without exudates | None | Occasional (1 or 2) airways affected; up to 5% of slide affected | Present in multiple airways; up to 25% of airways affected | Present in multiple airways; between 26-50% of airways  affected | Present in multiple airways; over 50% of airways affected |
| Peri-airway inflammatory infiltrates  (cuffing) | None | Occasional incomplete, or loosely formed cuffs; up to 5% of slide affected | Numerous cuffs; predominantly  incomplete and loosely formed  with lesser well-formed complete  cuffs; up to 25% of airways affected | Numerous cuffs; approximately  half or more well-formed, and  may have few broad, dense  cuffs; between 26-50% of airways affected | Numerous cuffs;  predominantly well-formed  with numerous broad, dense  cuffs; over 50% of airways affected |
| Perivascular inflammatory infiltrates  (cuffing) | None | Occasional incomplete, or loosely formed cuffs; up to 5% of slide affected | Numerous cuffs; predominantly  incomplete and loosely formed  with lesser well-formed complete  cuffs; up to 25% of vessels affected | Numerous cuffs; approximately  half or more well-formed, and  may have few broad, dense  cuffs; between 26-50% of vessels affected | Numerous cuffs;  predominantly well-formed  with numerous broad, dense  cuffs; over 50% of vessels affected |
| Alveolar walls / space infiltration by inflammatory cells, primarily neutrophils and macrophages;  variable oedema / fibrin +/- type II pneumocyte hyperplasia | None | Small numbers of foci affected within the parenchyma; up to 5% of the slide affected | Increased frequency of foci; between 6-25% of the slide affected | Multifocal coalescing or larger patches of parenchyma;  between 26-50% of the slide  affected | Large areas of parenchyma;  over 50% of the slide affected |
